# Relapsed Gray Zone Lymphoma: A Rare Clinical Challenge

**DOI:** 10.7759/cureus.65592

**Published:** 2024-07-28

**Authors:** Pratyaksh Chhabra, Prerna Sahu, Ishaan M Deshmukh, Prakriti Sharma, Bhushan Madke

**Affiliations:** 1 Pathology, Jawaharlal Nehru Medical College, Datta Meghe Institute of Higher Education and Research, Wardha, IND; 2 Dermatology, Venereology, and Leprosy, Jawaharlal Nehru Medical College, Datta Meghe Institute of Higher Education and Research, Wardha, IND

**Keywords:** hodgkin lymphoma, chemotherapy-related toxicity, adjuvant radiation therapy, diffuse large b lymphoma, gray zone lymphoma

## Abstract

Gray zone lymphoma (GZL) is an extremely uncommon hematological cancer with characteristics in common with both classical Hodgkin's lymphoma (HL) and diffuse large B-cell lymphoma. In this case report, we present a rare case of a 22-year-old male who developed HL initially and then transitioned to relapsed or refractory GZL. The disease relapsed twice throughout, even after the peripheral blood stem cell transplant. The initial biopsy suggested it was classical HL, and the biopsy was done late in the progression of the disease; the features suggested it was GZL. The treatment consisted of curative and preventive chemotherapy, radiation therapy, and immunotherapy.

## Introduction

Neoplasms arising from abnormal clonal proliferation of T-cells, B-cells, or natural killer cells are collectively known as lymphomas [1]. The two primary categories are Hodgkin's lymphomas (HLs) and non-HLs, with non-HLs comprising approximately 90% of all cases [2]. HLs are characterized by Reed-Sternberg cells, whereas non-HLs encompass a varied range of histological types [3]. Diffuse large B-cell lymphoma (DLBCL) is the most common type worldwide, characterized by the diffuse growth of large B-cells with enlarged nuclei. DLBCL is clinically aggressive with variable feedback to therapy [4]. Gray zone lymphoma (GZL) is a term in the 2022 World Health Organization classification for cases that do not fit into the HL or non-HL categories, bridging features of classical HL, nodular sclerosis subtype, and primary mediastinal B-cell lymphoma (PMBL). As early as 1998 and more frequently in 2005, GZL was first reported, gaining recognition from the World Health Organization in 2008. This lymphoma poses a significant challenge in both diagnosis and clinical management; pathologists encounter difficulties due to its complex morphological and phenotypic features, while oncologists struggle with its aggressive clinical behavior and limited treatment protocols defined by guidelines [5].

## Case presentation

A 22-year-old male came to the emergency department of a tertiary care center with a chief complaint of shortness of breath from the past few weeks. The patient was a frequent smoker and also had a history of alcohol consumption four years ago, and then he quit. There was no history of weight loss. Clinical examination findings were not notable except for lymphadenopathy. In the physical examination, the inguinal lymph nodes were enlarged, firm in consistency, immobile, and approximately 3-4 cm in size. There were no other significant findings in the clinical examination, and no relevant issues were found in his medical history.

**Figure 1:**
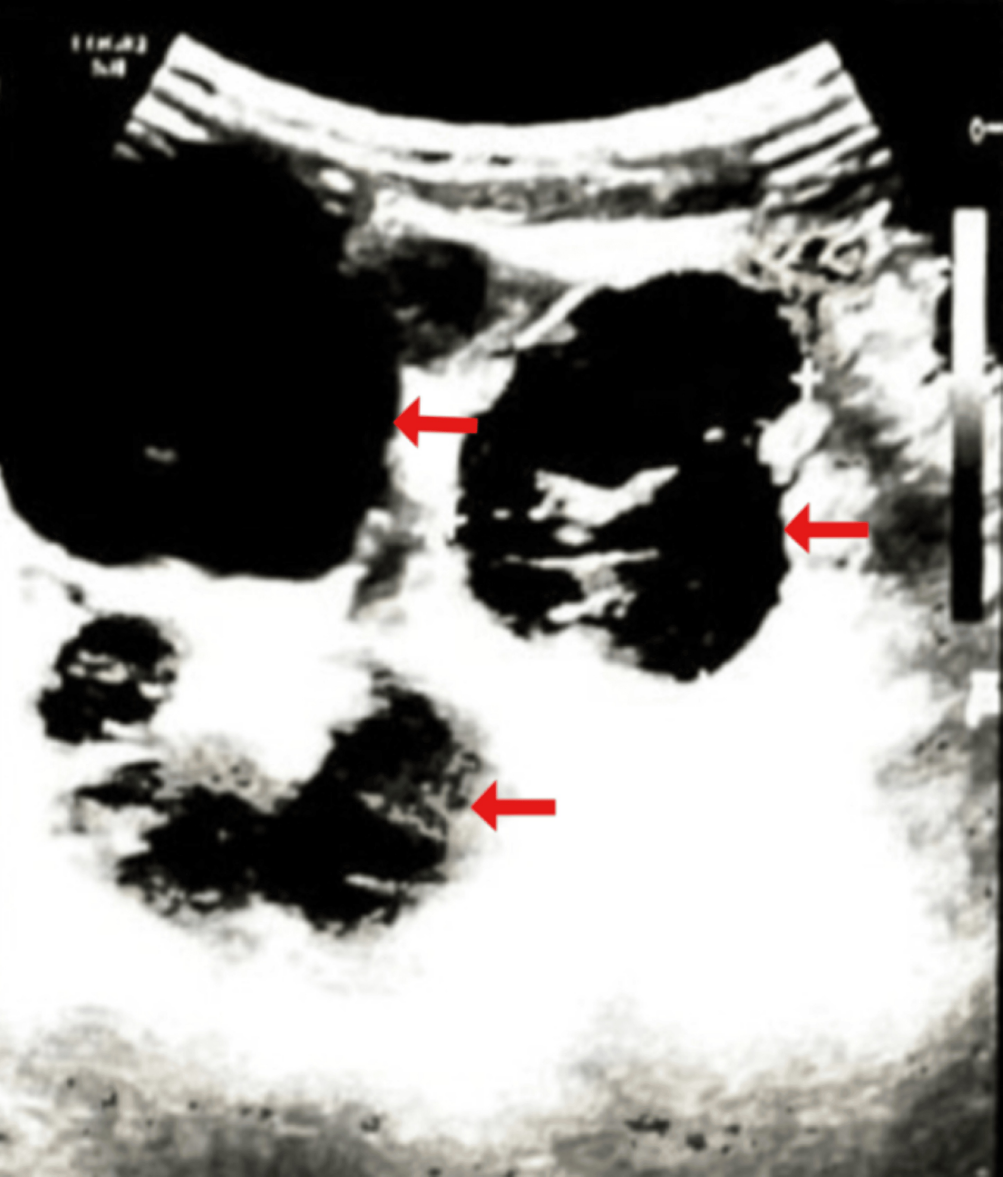
USG showing multiple enlarged round to oval lymph nodes that were found extending from the left side of the aortic bifurcation along the iliac vessels up to the inguinal region USG: ultrasonography

A pulmonary embolism was detected on CT angiography and was treated. A Doppler ultrasonography (USG) scan of the lower limbs and the inguinal region was done, which showed multiple enlarged round to oval lymph nodes extending from the left side of aortic bifurcation along iliac vessels up to the inguinal region (Figure 1). The lymph nodes were 4.5-5 cm in size. A left inguinal lymph node histopathologic examination and medium biopsy were done in April 2022, and the possibility of HL was suggested.

**Figure 2:**
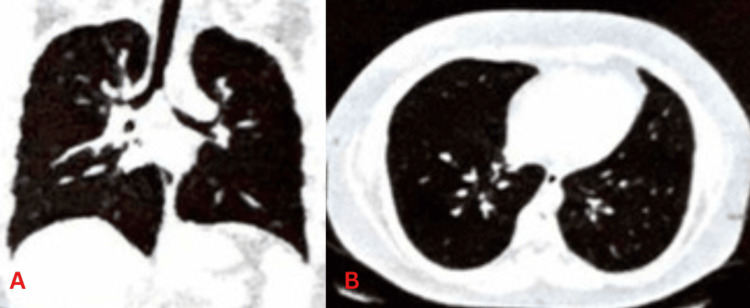
HRCT chest scan showing multiple centrilobular nodules scattered in the bilateral lungs A: coronal view, B: axial view, HRCT: high-resolution computed tomography

The surgical excision was done for enlarged inguinal lymph nodes in April 2022, whose features favored malignant lymphoma. Immunohistochemistry (IHC) of inguinal lymph nodes showed T-cell histiocyte-rich B-cell lymphoma-like transformation of nodular lymphocytes, predominant in classic HL. Chemotherapy was started in May 2022 after the left inguinal lymph node surgical excision. The regimen was six cycles of rituximab, doxorubicin, bleomycin, vinblastine, and dacarbazine (R-ABVD) given six hours every two weeks. After the fourth cycle, bleomycin was stopped due to drug-induced lung injury/adverse effects. A high-resolution CT (HRCT) chest scan was done, and it showed multiple centrilobular nodules scattered in bilateral lungs (Figure 2).

**Figure 3:**
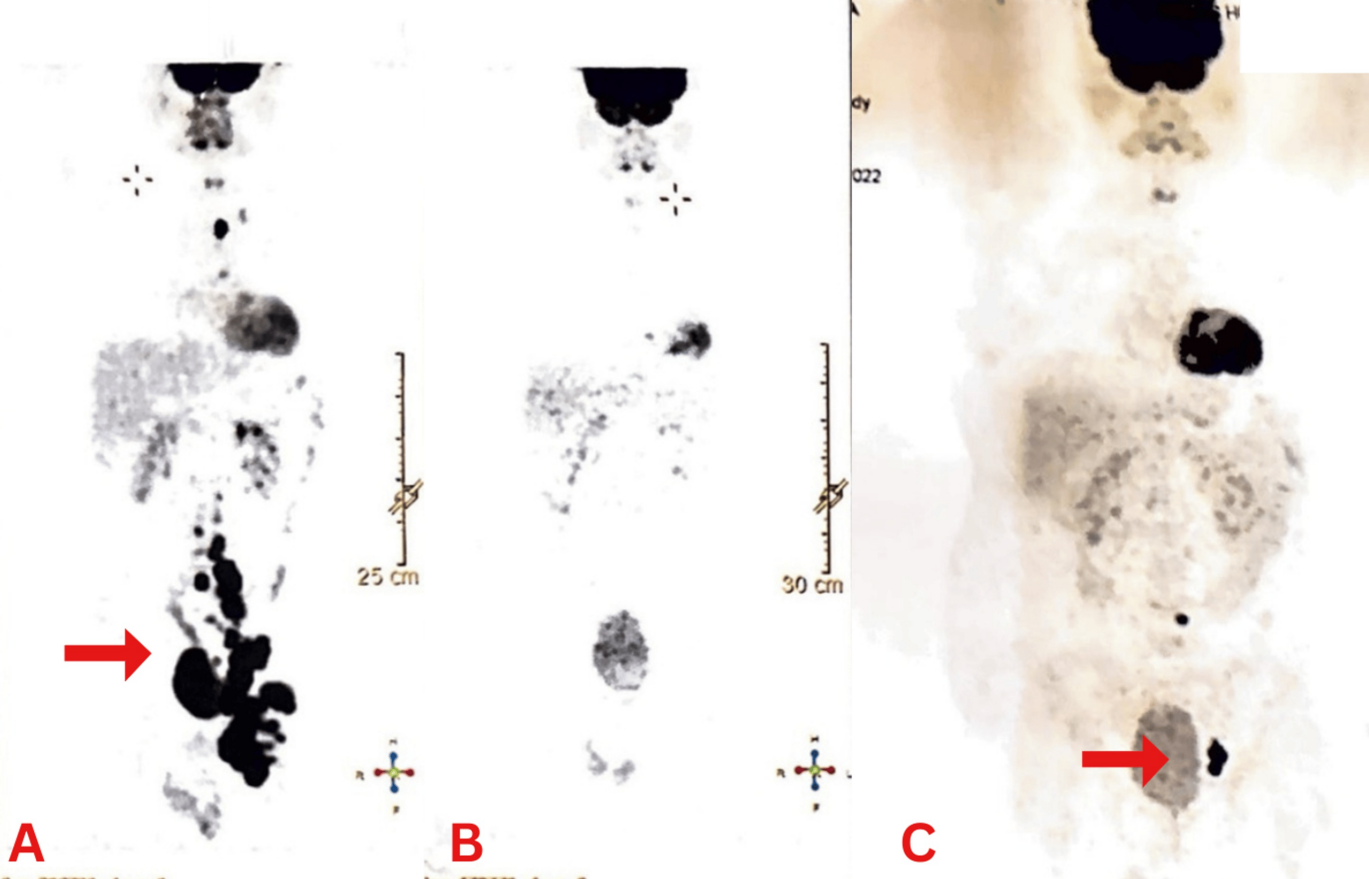
PET CT scans. (A) Large FDG avid retroperitoneal and pelvic lymphadenopathy. (B) Clear scan suggesting a favorable prognosis. (C) Appearance of new multiple FDG avid retroperitoneal, left iliac, and left femoral lymph nodes PET: positron emission tomography, CT: computed tomography, FDG: fluorodeoxyglucose

A positron emission tomography (PET) CT scan was done in May 2022, and it was suggestive of large 18F-fluorodeoxyglucose (FDG) avid retroperitoneal and pelvic lymphadenopathy (Figure 3A). Chemotherapy was continued with the fifth cycle in May 2022. The regimen was rituximab, doxorubicin, vinblastine, and dacarbazine (R-AVD), and bleomycin was removed due to drug reactions. In July 2022, a PET CT scan was done, and a favorable diagnosis was suggested (Deauville's score 1) (Figure 3B).

After six cycles of chemotherapy, in November 2022, a PET CT scan was done. It was suggestive of the interval appearance of new multiple FDG avid retroperitoneal, left iliac, and left femoral lymph nodes (Figure 3C). Overall scan findings likely suggested disease progression (Deauville's score 5a). The disease is now termed relapsed relapsed/refractory after the recurrence. It was decided to do an autologous peripheral blood stem cell transplant (PBSCT) post chemotherapy due to the recurrence and the aggressive nature of the disease variant.

**Figure 4:**
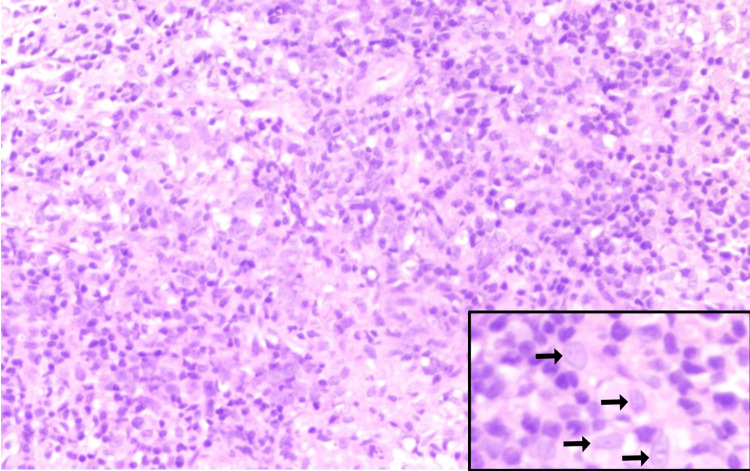
H&E-stained section showing multiple cores of lymphoid tissue with scattered large mononucleate to binucleate cells (inset) with a background composed predominantly of small to medium-sized lymphocytes H&E: hematoxylin and eosin

A biopsy of left inguinal lymph nodes was done in November 2022, and histopathology showed multiple cores of lymphoid tissue with scattered large mononucleate to binucleate cells. Some of them show vesicular nuclei and prominent eosinophilic nucleoli. The background is composed predominantly of small to intermediate-sized lymphocytes, occasional polymorphonuclear leukocytes, and immunoblasts. Some of the large cells show atypical mitotic figures. Some areas were also seen with marked crushing and ischemic necrosis (Figure 4).

**Figure 5:**
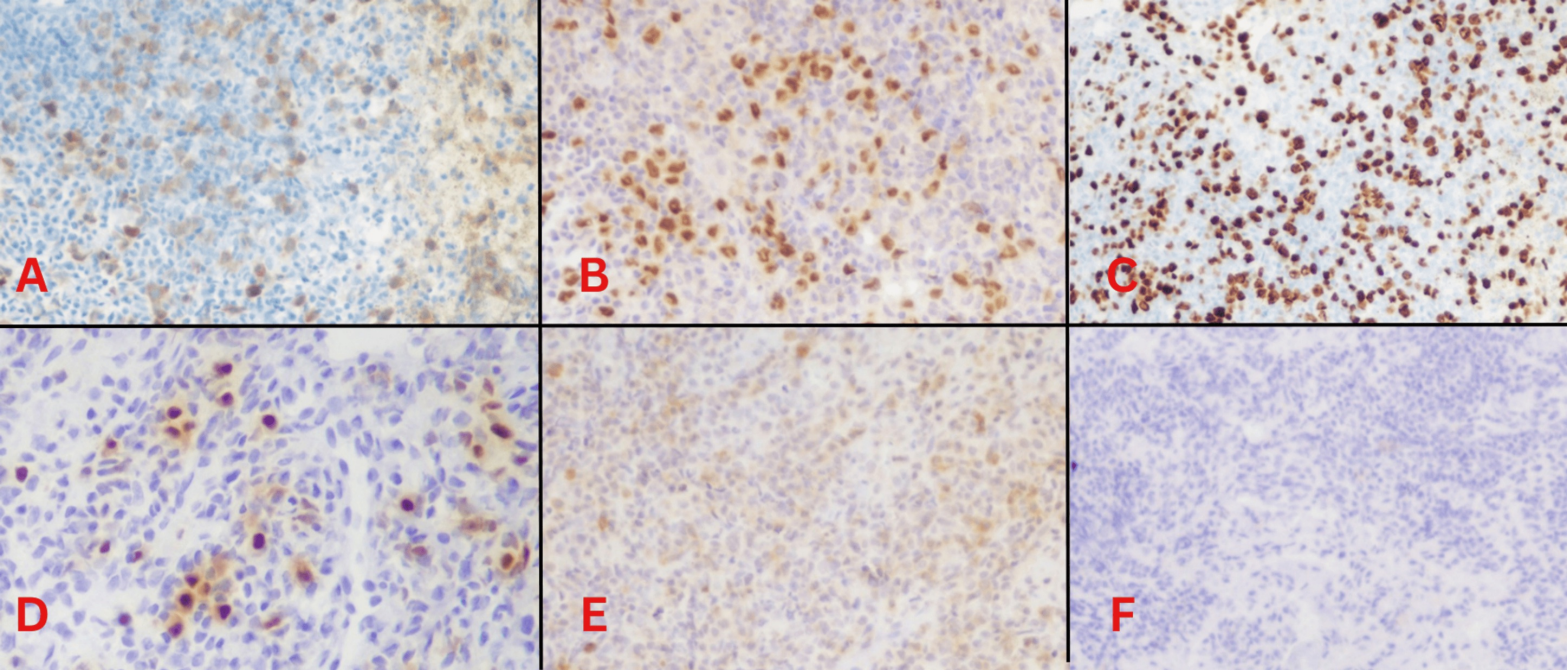
IHC showing positivity for (A) CD79A, (B) PAX5, (C) Ki-67 (60%), (D) MUM1, (E) CD30 and negative due to rituximab therapy, and (F) CD20

IHC showed the large cells express CD19, CD79A, PAX5, MUM1, OCT2, and BOB1. Expression for CD20 is lost due to rituximab therapy. CD30 stains a few large cells and also the bystander immunoblast population. CD45 staining is variable, with positivity seen in a few large cells and the remaining negative. The cells are also negative for EBERish, CD3, and CD15. Ki-67 stains about 60% of the overall cell population, which includes all the large cells (Figure 5). Features consistent with a GZL with features intermediate between classical HL and DLBCL.

After the first recurrence, chemotherapy was started in December 2022. The regimen was three cycles of rituximab, etoposide phosphate, prednisone, vincristine sulfate, cyclophosphamide, and doxorubicin hydrochloride (R-EPOCH).

A PET CT scan done in January 2023 was negative and showed no lesions (Deauville’s score 1). A PBSCT was done in February 2023 as per the treatment regimen that was decided before due to the recurrence and aggressive nature of the disease variant. Post PBSCT, a PET CT scan was done in May 2023, which was suggestive of no lesions and showed a complete response to therapy (Deauville’s score 1). Prevention chemotherapy was started thereafter with a regimen of 15 cycles of brentuximab every three weeks.

**Figure 6:**
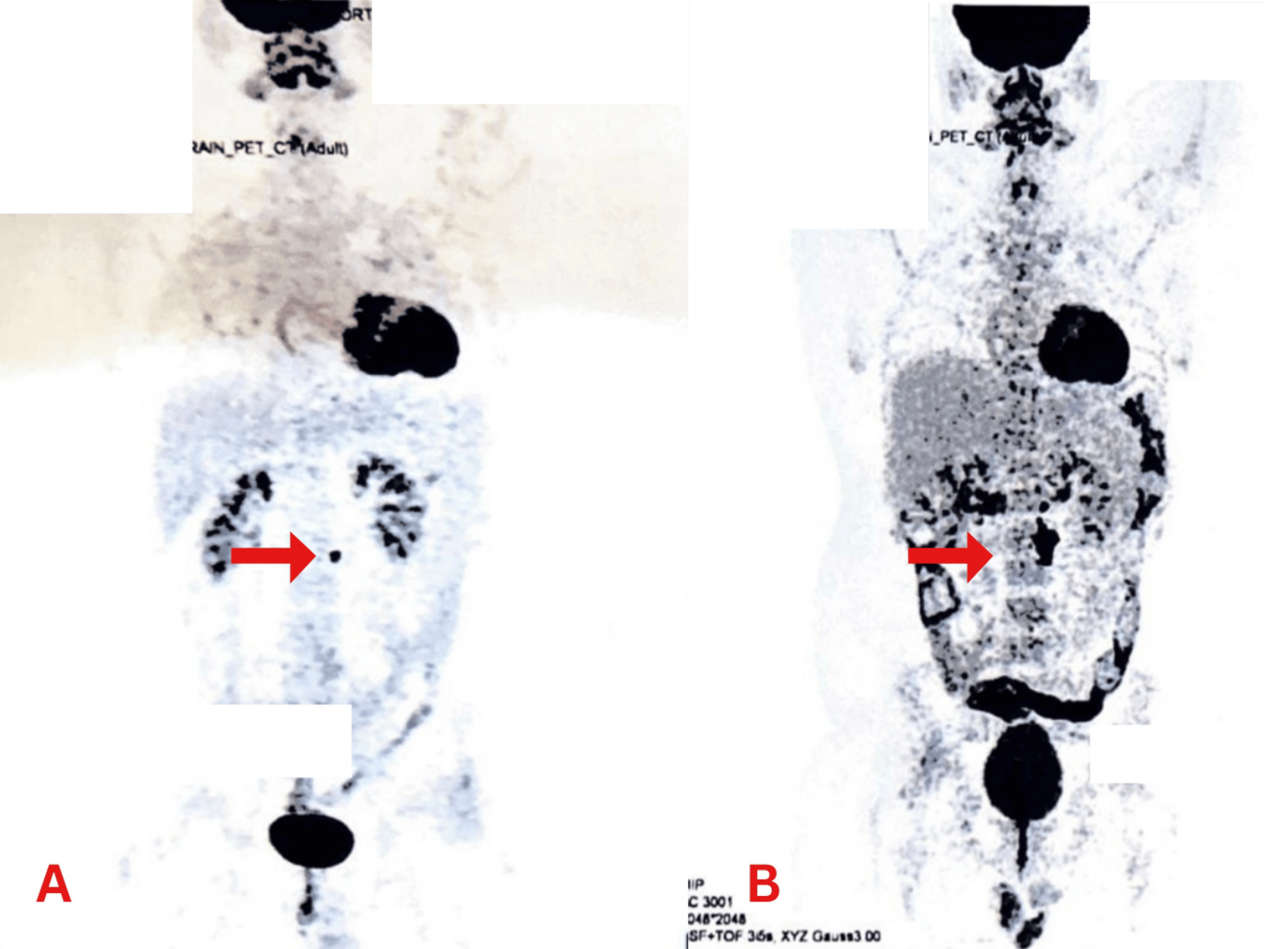
PET CT scans. (A) Scan shows a tiny FDG avid lymph node in the para-aortic region. (B) Scan suggestive of an increase in size and number of FDG avid lymph nodes in the para-aortic region, likely representing disease progression PET: positron emission tomography, CT: computed tomography, FDG: fluorodeoxyglucose

As a follow-up, a PET CT scan done in August 2023, post chemotherapy and PBSCT, was suggestive of an FDG avid tiny lymph node in the para-aortic region. The patient was asked to follow up (Figure 6A). A PET CT scan done in November 2023 is suggestive of an increase in the size and number of FDG avid lymph nodes in the para-aortic region, likely representing disease progression (Deauville's score 5) (Figure 6B). The disease recurred again for the second time.

In November 2023, a retroperitoneal lymph node biopsy was done and was suggestive of large B-cell lymphoma, favoring T-cell-rich B-cell lymphoma. Chemotherapy was started after the second recurrence. The regimen was five cycles (three curative + two preventive post radiation therapy) of rituximab, gemcitabine, dexamethasone, and cisplatin (R-GDP) every three weeks. A PET CT scan done in January 2024 was suggestive of complete remission (Deauville’s score 1).

Radiation therapy was started in February 2024. The regimen was 16 cycles. After the initial nine cycles, the radiation therapy was stopped due to a lung infection with coronavirus. After the infection cleared, the remaining seven cycles of radiation therapy were given. The site was para-aortic, and left inguinal lymph nodes and image-guided radiation therapy were given.

Post radiation preventive chemotherapy was started. The regimen consisted of the remaining two cycles of R-GDP every three weeks. Other drugs were also given orally each time to prevent adverse side effects of the therapy. Currently, the patient is on immunotherapy. The regimen is nivolumab every two weeks. Also, rituximab is given every four weeks as a preventive measure for recurrence.

## Discussion

Since GZL is extremely rare, an accurate estimation of its incidence is impossible. Nevertheless, Qasrawi et al. used age-adjusted incidence rates based on cases that were confirmed between 2005 and 2016 and the US Standard Population in 2000 to estimate incidence at a rate of 0.53 per million person-years [6]. Furthermore, GZL was further classified as mediastinal GZL (MGZL) and non-MGZL in 2008 [7,8].

Reed-Sternberg cells, which are characteristic of the disease, will increase the probability that classic HL is the diagnosis [8]. The B-cell-related transcription factors BOB.1 and OCT.2, whose involvement in immunoglobulin regulation is prominently expressed in PMBL but missing in classic HL, are further indicators to distinguish between PMBL and classic HL. While CD30 is expressed in both cases, it is more variable and weaker in PMBL than in traditional HL [9]. Furthermore, several genes specific to PMBL, such as MAL, CD23, TARC, NFkB2, and PDL1/L2, are expressed. From that point on, GZL was defined to represent the "gray area" of diagnosis when it came to identifying either PMBL or classic HL that exhibited a morphologically characteristic presentation but an immunophenotypically uncharacteristic presentation, lacking CD20 or strongly expressing CD15 [7-9].

MGZL's histological composition ranges from that of PMBL to that of typical HL with nodular sclerosis. In verified cases of GZL, the morphological composition of the cells is defined as sheet-like, with a high degree of pleomorphism expressed by the tumor cells and a diffusely appearing tumor architecture that may be coarsely fibrotic or nodular [9,10].

GZL therapy options have typically been comparable to PMBL treatment options [10]. This is partially caused by the absence of established recommendations, which is probably connected to the disease's rarity and difficult diagnosis. In 2004, the National Institutes of Health initiated prospective research on dose-adjusted etoposide, prednisone, doxorubicin, cyclophosphamide with vincristine, and rituximab (DA-EPOCH-R), which resulted in the first documented therapy of 24 confirmed instances of MGZL that had not been treated before. Event-free survival and overall survival were 62% and 74%, respectively, over the median follow-up of 59 months [7,11].

Relapsed/refractory GZL denotes a more focused, conclusive course of treatment. Studies like Evens et al., which had 112 individuals treated with different therapeutic regimens and resulted in 65 relapses with a median period of seven months before moving on to salvage therapy, have historically used chemotherapy salvage regimens. ABVD (n=2), gemcitabine-based (n=3), etoposide, solu-medrol, cytarabine, and cisplatin (n=8), and ifosfamide, carboplatin, and etoposide (n=39) were among the regimens examined. Four individuals were treated with biologic therapy, which included brentuximab [7,12].

Although GZL is, by definition, categorized as existing between two other types of cancer, it should be considered a distinct entity. This differentiation is important only from a therapeutic standpoint rather than necessarily from a diagnostic one [7].

## Conclusions

The histopathological diagnosis and classification of GZL are difficult and can vary between observers. Nonetheless, accurate identification and diagnosis are essential for starting the best possible treatment. Very few case reports have been published so far regarding GZL, underscoring the need for thorough study in this field. The rare incidence of this disease makes it an area for research in the future. There is no specific treatment protocol due to the very low occurrence of this cancer. It is very critical to identify the transition from one type of lymphoma to another. The diagnosis and therapeutic interventions are both very crucial in determining the progression of the illness.
